# Soft-Landing Dynamic Analysis of a Manned Lunar Lander Em-Ploying Energy Absorption Materials of Carbon Nanotube Buckypaper

**DOI:** 10.3390/ma14206202

**Published:** 2021-10-19

**Authors:** Qi Yuan, Heng Chen, Hong Nie, Guang Zheng, Chen Wang, Likai Hao

**Affiliations:** 1 State Key Laboratory of Disaster Prevention & Mitigation of Explosion & Impact, Army Engineering University of PLA, Nanjing 210007, China; yuanqi1900@126.com; 2 College of Field Engineering, Army Engineering University of Chinese People's Liberation Army, Nanjing 210007, China; 3 College of Aerospace Engineering, Nanjing University of Aeronautics and Astronautics, Nanjing 210016, China; hnie@nuaa.edu.cn; 4 Faculty of Mechanical Engineering and Mechanics, Ningbo University, Ningbo 315000, China; zhengguang@nbu.edu.cn; 5 Key Laboratory of Exploration Mechanism of the Deep Space Planet Surface, Ministry of Industry and Information Technology, Nanjing University of Aeronautics and Astronautics, Nanjing 211100, China; nuaawangchen@nuaa.edu.cn; 6 Unit 66133 of Chinese People's Liberation Army, Beijing 100000, China; hao_likai@163.com

**Keywords:** CNT buckypaper, soft-landing, multibody dynamic models, energy absorption, coarse-grained molecular dynamics simulations

## Abstract

With the rapid development of the aerospace field, traditional energy absorption materials are becoming more and more inadequate and cannot meet the requirements of having a light weight, high energy absorption efficiency, and high energy absorption density. Since existing studies have shown that carbon nanotube (CNT) buckypaper is a promising candidate for energy absorption, owing to its extremely high energy absorption efficiency and remarkable mass density of energy absorption, this study explores the application of buckypaper as the landing buffer material in a manned lunar lander. Firstly, coarse-grained molecular dynamics simulations were implemented to investigate the compression stress-strain relationships of buckypapers with different densities and the effect of the compression rate within the range of the landing velocity. Then, based on a self-designed manned lunar lander, buckypapers of appropriate densities were selected to be the energy absorption materials within the landing mechanisms of the lander. For comparison, suitable aluminum honeycomb materials, the most common energy absorption materials in lunar landers, were determined for the same landing mechanisms. Afterwards, the two soft-landing multibody dynamic models are established, respectively, and their soft-landing performances under three severe landing cases are analyzed, respectively. The results depicted that the landers, respectively, adopting the two energy absorption materials well, satisfy the soft-landing performance requirements in all the cases. It is worth mentioning that the lander employing the buckypaper is proved to demonstrate a better soft-landing performance, mainly reflected in reducing the mass of the energy absorption element by 8.14 kg and lowing the maximum center-of-mass overload of the lander by 0.54 g.

## 1. Introduction

The moon is not only the closest celestial body to the earth, but also the only natural satellite of the earth. Due to its potential resources and unique space location, the moon has become the preferred target for humanity in carrying out deep space exploration. During the lunar exploration boom from 1958 to 1976, a total of 108 lunar probes were launched by the United States and the former Soviet Union. The most eye-catching one was the “Apollo 11” lunar manned probe, which succeeded in manned soft landing on the moon for the first time [1,2,3,4]. After years of silence, China’s “Chang’e Project” also successfully realized the soft landing of unmanned lunar landers on the moon three times from 2014 to 2020 [5,6,7]. Soft landing means that the maximum center-of-mass acceleration of the lander is limited within several g to a dozen g during the whole landing process. The part that lands on the moon’s surface is usually called the lander. For both the “Apollo” series of landers and the “Chang’e” series of landers, the landing overloads were controlled within a small range through the soft-landing mechanisms, thereby protecting the integrity of the structure and equipment and the safety of astronauts [8,9]. For all of those landers, impact energy was mainly dissipated by aluminum honeycomb element within the landing mechanisms by means of plastic collapse [10,11,12,13,14]. Aluminum honeycomb, a kind of porous metal material, is a new type of physical engineering material which was developed rapidly in the late 1980s. Due to its excellent physical properties and deformation characteristics of plastic collapse, aluminum honeycomb has been widely used in the fields of shock absorption and energy absorption, and the mass density of energy absorption is about 15–20 J/g [15,16,17,18,19]. With the sharp pace of deep space exploration, such as further manned lunar exploration, the establishment of lunar bases, the development and utilization of lunar resources, etc., the mass of the lander will be greatly increased. Traditional energy absorption materials, even including aluminum honeycomb, are becoming more and more inadequate in meeting the increasing requirements of light weight, high energy absorption efficiency, and high energy absorption density. Therefore, developing novel energy absorption materials is a matter of a great urgency.

In recent years, the randomly distributed CNT network, also called CNT buckypaper, has attracted wide attention [20,21,22,23,24,25,26,27]. In a buckypaper, due to the long range of van der Waals (vdW) interaction between carbon atoms, CNTs gradually aggregate to form bundles or entanglements [28,29,30]. L. Zhang and S.W. Cranford et al. [31,32] found that the Young’s modulus of buckypaper could be adjusted from 0.2 GPa to 3.1 GPa by changing the density and the diameter of CNTs; L.J. Hall et al. [29] found that the Poisson’s ratio of buckypaper could be adjusted between positive and negative values by changing the content of multi-walled CNTs. Studies by R.L.D. Whitby et al. [33] showed that a buckypaper usually had a very low density (0.05–0.4 g/cm^3^) and high porosity (0.8–0.9); Q. Wu et al. [34] found that the pore size of a buckypaper constructed by single-walled CNTs with diameters of 0.8–1.2 nm and lengths of 100–1000 nm was about 10 nm. What is more noteworthy is that, Y. Li and M. Xu et al. [35,36,37] revealed the viscoelastic characteristics of buckypaper by experiments and simulations, especially the frequency independence and temperature independence in the temperature range of −196−1000°. Additionally, the mechanism of energy dissipation was found to be the zipping-unzipping behavior between CNTs [35,36]. Our previous work [38,39] also indicated that the buckypaper possessed a great capacity of plastic deformation; the energy absorption efficiency could reach up to 98%, the mass density of energy absorption could reach up to 90 J/g (much higher than that of aluminum honeycomb), and by increasing the density of buckypaper, the mass density of energy absorption could be further improved.

All of these results show that buckypaper possesses distinguished energy absorption characteristics and crashworthiness, which makes it a potential candidate for energy absorption materials of future lunar landers [40,41,42]. Hence, this paper explores the possibility of the application of buckypapers in a manned lunar lander. Firstly, coarse-grained molecular dynamics simulations are implemented to study the uniaxial compression mechanical properties of buckypaper with different densities. Then the corresponding stress-strain relationships are obtained, and the influence of compression rate within the range of landing velocity is analyzed. Secondly, for comparison, suitable buckypapers and aluminum honeycombs are, respectively, determined as the energy absorption materials within the landing mechanisms of a self-designed manned lunar lander. For convenience, the lander adopting buckypapers is calleda buckypaper-buffering lander and the lander adopting aluminum honeycomb is called an aluminum honeycomb-buffering lander. Then, soft-landing dynamical models of both the two landers are established and three severe landing cases are selected. Finally, for all the three cases, the soft-landing performances of the two landers are analyzed, respectively.

## 2. Model and Characteristics of Buckypaper

### 2.1. Coase-Grained Molecular Dynamic Model

In this work, a buckypaper is built of (5,5) single-walled carbon nanotubes (SWCNTs). A coarse-grained molecular dynamics (CGMD) method is applied to establish large-scale CNT buckypapers. In the CGMD model, each CNT is simplified as a multi-bead chain. For a particular chain, stretching properties can be described by bonds between two adjacent beads, and bending properties can be described by angles among three successive beads. The interaction between different CNTs can be expressed by long-ranged (vdW) interaction between pairs of beads. The total energy of this CGMD model can be expressed as follows:

(1) $$E_{tot}=E_{bond}+E_{angle}+E_{vdW}$$

where $E_{bond}=\sum_{bonds}\frac{1}{2}k_{b}{\left(r-r_{0}\right)}^{2}$ is the inter-chain stretching energy. Buehler and Cranford [43,44,45] have validated that $r_{b0}$, $\theta_{0}$, and  σ  can be determined by equilibrium conditions, and $k_{b}$, $k_{a}$, and ε can be determined by the principle of conservation of energy. In detail, the stretching behavior of a CNT can be described by the uniaxial tension tests and then the Young’s modulus E can be obtained. Thus $k_{b}$ can be calculated by $EA/r_{0}$, where A is the cross-sectional area of the CNT. Bending tests can determine the bending behavior and bending stiffness EI (I is the bending moment of inertia) of the CNT and thus $k_{a}$= $3EI/2b_{0}$ can be gained. Simulations of an atomistic assembly of two CNTs can be implemented to calculate the equilibrium distance D between them and their adhesive strength β, and thereby ε and σ can be acquired via $\varepsilon =\beta r_{0}$ and $\sigma =D/\sqrt[{6}]{2}$, respectively. The relevant parameters of the coarse-grained model in this work are shown in Table 1.

### 2.2. Structure of (5,5) SWCNT Buckypaper

**Figure 1:**
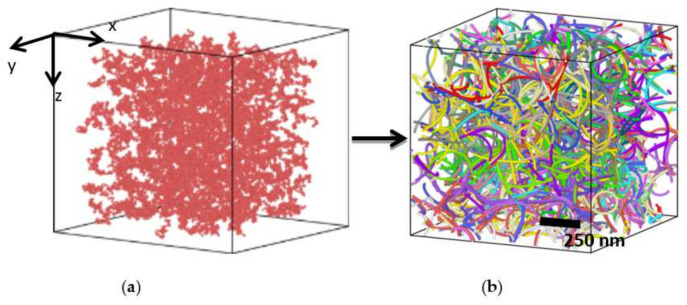
Establishment of a CGMD of buckypaper. (**a**) Random walk; (**b**) Buckypaper.

A random walk method is adopted to generate the structure of buckypaper to ensure the randomness and isotropy. Initially, a series of points are put into an orthogonal cell in a spatially uniform manner, serving as the positions for the initial beads of SWCNTs. Each SWCNT grows to the next position for the next bead by a random bond vector with the magnitude equal to the equilibrium bond length (10 Å). Specifically, when the distance between the newly generated bead and its nearest bead is less than a specific distance 2 Å, it is considered that the position of the newly generated bead has been occupied by other beads, so the CNT returns to the position of the previous step, re-generates a random bond vector, and generates a new bead position. The process of coincident inspection needs to be repeated until the new bead position is not occupied by other beads, and then the new bead is formed. When the random walk distance is equal to the desired length of a single CNT, the walk ends, and the current bead is the end bead of this CNT. In this work, all the CNTs in a box have the same length. After the energy minimization and equilibrium process, the equilibrium state of a buckypaper can be obtained, as shown in Figure 1. All three axes of the simulation box are set to be periodic so that a large-scaled buckypaper with continuous mass can be generated. The establishment of the CGMD model of a buckypaper is described in more detail in our published papers [38,39].

### 2.3. Compression Characteristics of Buckypaper

**Figure 2:**
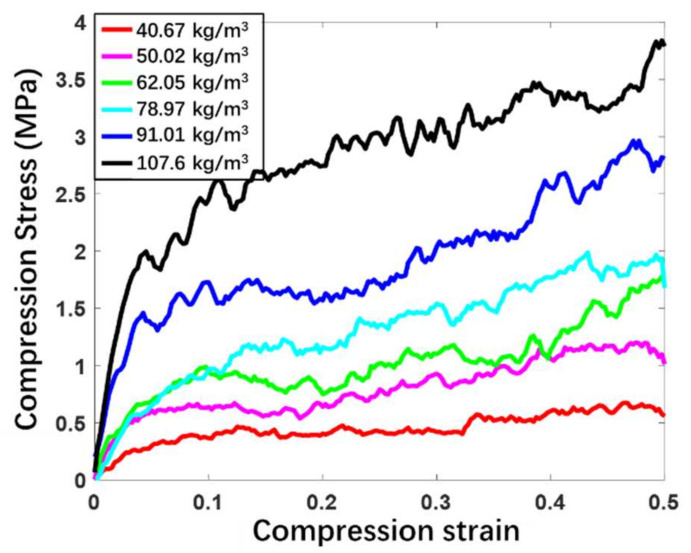
Compression stress as a function of compression strain for buckypapers with different densities.

**Figure 3:**
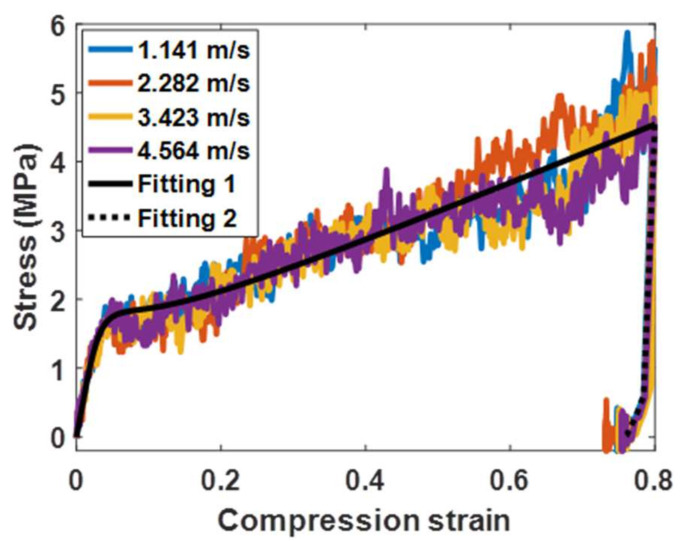
Compression stress as a function of compression strain for the buckypaper with density of 107.6 kg⁄m^3^ under different compression rate.

The uniaxial compression simulations of buckypapers with different densities are performed on the open source platform LAMMPS [46]. The deformation of the buckypaper is realized by controlling the displacement of the simulation box in the x direction, allowing the y and z directions to fluctuate. The compression ratio is $10^{8}/s$ and the time step is 10 fs. The compression stress $\;\sigma_{x}$ in the x direction can be calculated by virial formula. The compression strain $\varepsilon_{x}$ is given by $1-L_{x}/L_{x0}$, where $L_{x}$ and $L_{x0}$ are the current length and initial length of the buckypaper in the x direction, respectively. Similarly, the lateral propagation strains are, respectively, defined as $\varepsilon_{y}=L_{y}/L_{y0}-1$ and $\varepsilon_{z}=L_{z}/L_{z0}-1$,where the subscripts y and z represent the corresponding directions, respectively. According to the initial linear stage, the Young’s modulus E can be calculated by $E=-\sigma_{x}/\varepsilon_{x}$. Compression stress-strain relationships of buckypapers with six different densities are obtained by related compression simulations and demonstrated in Figure 2. It can be seen that the Young’s modulus of the buckypaper increases with the increase of its density. Since the vertical landing velocity of the lunar lander is generally less than 4 m/s, in order to understand the influence of compression rate on the stress-strain relationship of the buckypaper, four different compression rates in the 0–4 m/s range (1.141 m/s,  2.282 m/s, 3.423 m/s and 4.564 m/s) are imposed on the buckypaper with a density of $107.6\;\mathrm{kg}/m^{3}$, and the corresponding relationships between compression stress σ and compression strain ε are depicted in Figure 3. It can be seen that the compression strain rate has no obvious effect on the stress-strain relationship within the specific rate range. Therefore, the influence of the landing velocity on the relationship between cushioning force and cushioning stroke can be ignored. Additionally, for the strain less than 3%, the buckypaper is in the elastic stage and the compression stress increases rapidly with the increase of the compression strain. However, for the strain over 3%, the compression stress of the buckypaper increases slowly with the increase of strain. Generally, the effective cushioning stroke of the buckypaper can reach about 80%. It can be inferred that after the compaction of the buckypaper, the growth of the compression stress will become sharp. When the compression load is removed, the buckypaper will rebound rather slightly with a high level of non-recoverable deformation, resulting in a considerable amount of energy dissipation. The area surrounded by the compression stress-strain curve in Figure 3 is just the energy absorption $E_{I}$ by the buckypaper per unit volume (that is, $E_{I}=\int \sigma d\varepsilon$).

## 3. Dynamic Model and Computational Methods of Soft Landing

### 3.1. Overall Scheme

**Figure 4:**
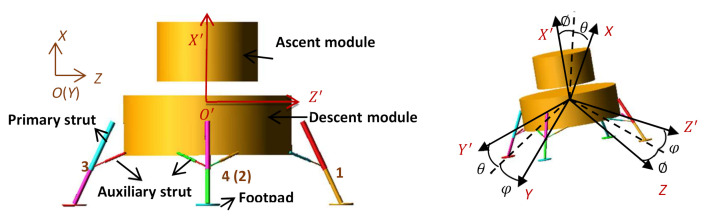
Overall Scheme of a manned lunar lander.

The overall scheme of a manned lunar lander is shown in Figure 4. It consists of a main body (including an ascent module and a descent module) and four landing mechanisms (also called landing legs). The four landing mechanisms are identical and evenly arranged. In order to improve the computational efficiency, it is necessary to simplify those components that have few influences on landing performance. For example, the main body is considered to be two connected cylinders both with height of 2.5 m, maintaining characteristics of mass and moment of inertia. Each landing gear system is comprised of one primary strut, two auxiliary struts, and one footpad. Both the primary and auxiliary struts contain an outer cylinder and an inner cylinder. The primary strut is attached to the module at the upper end of the outer cylinder by a universal fitting and to the pad at the lower end of the inner cylinder by a ball fitting. Within the primary strut is a compression buffer component which is made of energy absorption materials. The auxiliary strut is attached to the module at the upper end of the outer cylinder by a universal fitting and to the primary strut at the lower end of the inner cylinder by a ball fitting. The auxiliary strut, having a bidirectional buffer capability, contains two buffer components which are made of the same energy absorption materials. Sliding between the outer cylinder and inner cylinder causes the compression of related buffer components, thus absorbing impact energy by plastic deformation. The dish-shaped footpad is allowed to rotate while sliding, preventing the lander from sinking to the soft lunar surface excessively and improving the landing stability in case the initial horizontal velocity is fairly large. The modules, primary and auxiliary struts except buffer components, and footpads can be roughly treated to be rigid.

### 3.2. Definitions of the Coordinate Systems

As shown in Figure 4, two different right-handed Cartesian systems are employed to establish the soft landing dynamic model. They are inertial coordinate system and body coordinate system, which are defined as follows.

(1) In inertial coordinate system *OXYZ*, the origin *O* is located in initial barycenter of the main body and fixed to the lunar surface, *X* axis is direct to the opposite of lunar gravity. *Z* axis is perpendicular to *X* axis and direct to downhill. *Y* axis is determined by the rule of right-handed Cartesian system.

(2) In body coordinate system $O^{\prime }X^{\prime }Y^{\prime }Z^{\prime }$, the origin $O^{\prime }$ is located in barycenter of the main body. $X^{\prime }$ axis is perpendicular to the interface of the lunar lander and carrier rocket and point up. $Z^{\prime }$ axis is perpendicular to $X^{\prime }$ axis and direct to Leg 1. $Y^{\prime }$ axis is determined by the rule of right-handed Cartesian system.

Other landing legs are numbered in turn in counterclockwise direction from top view. The auxiliary struts are numbered in form of “i-j”. Here, “i” is the number of the corresponding landing leg. “j” equals “1” for the left auxiliary strut and “2” for the right one from outside view.

(2) $$T_{ib}=T_{bi}^{T}=\left[\begin{array}{ccc} \cos\theta \cos\emptyset & -\sin\theta \cos\varphi +\cos\theta \sin\emptyset \sin\varphi & \sin\theta \sin\emptyset +\cos\theta \sin\emptyset \cos\varphi \\ \sin\theta \cos\emptyset & \cos\theta \cos\varphi +\sin\theta \sin\emptyset \sin\varphi & -\cos\theta \sin\varphi +\sin\theta \sin\emptyset \cos\varphi \\ -\sin\emptyset & \cos\emptyset \sin\varphi & \cos\emptyset \cos\varphi \end{array}\right]$$

### 3.3. Determination of Key Geometric Parameters

**Figure 5:**
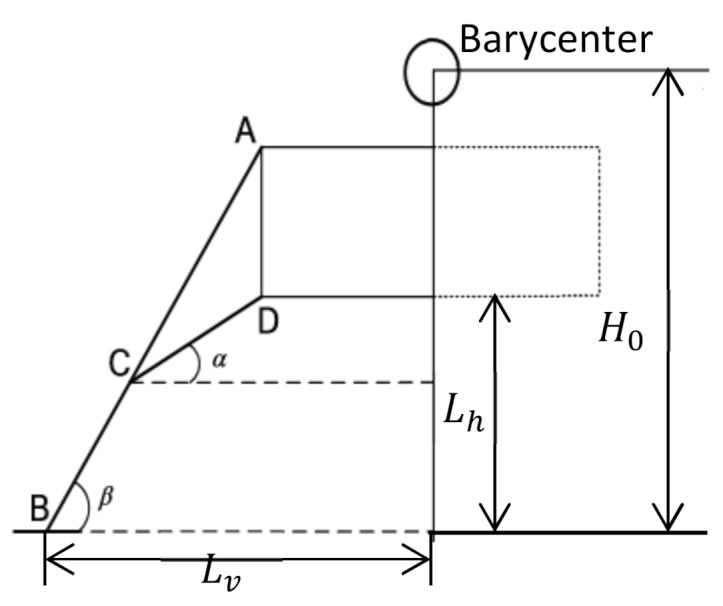
Compression stress as a function of compression strain for the buckypaper with density of 107.6 kg⁄m^3^ under different compression rate.

There are two important geometric parameters of landing legs. As shown in Figure 5, One is the initial height from the body’s bottom to the local lunar surface, expressed by $L_{h}$. The other is the distance between the line connecting the centers of two adjacent footpads and the central axis of the body, expressed by $L_{v}$. Since the surface of the moon is strewn with large and small rocks and other bumps, the bottom of the body is necessary to keep a safe distance from the lunar surface during the whole landing process, avoiding contacting the bumps. That means $L_{h}$ should not be too small. However, $L_{h}$ should not be too large. Otherwise, the initial distance between the barycenter and the lunar surface, expressed by $H_{0}$, could be too large, reducing landing stability. Landing stability is related to the “stability polygon” which is defined as a regular polygon whose vertexes are the centers of four footpads. It can be roughly believed that during the landing process, if the projection of the barycenter of the lander along the direction of gravity is located inside the stability polygon, the landing process is stable, that is, the lander will not tip over. As can be seen from Figure 5, increasing $L_{v}$ can increase the area of the stability polygon, so as to improve the landing stability of the lander. However, the increase of $L_{v}$ also means the increase of the length of both primary and auxiliary struts, thus increasing the mass of the landing legs. Therefore, $L_{h}$ and $L_{v}$ affect the landing stability and the mass of the landing legs simultaneously. On the premise of ensuring landing stability, in order to reduce the mass of the lander, $L_{h}$ and $L_{v}$ should be as small as possible.

The minimum value of $L_{h}$ can be estimated by the following formula:

(3) $$L_{h,min}\approx S_{h}+S_{0}+∆H$$

where, $S_{h}$ is the displacement of the primary strut in the vertical direction, $S_{0}$ is the safe distance between the bottom of the lander and the lunar surface after landing, and ∆H is the subsidence displacement of the barycenter of the lander relative to the lunar surface during landing.

(4) $$H_{0}=L_{h,min}+L_{0}$$

In order to ensure that the lander does not tip over during landing, the following condition must be met:

(5) $$W_{D}\leq W_{H}$$

where, $W_{D}$ is the kinetic energy of the lander at the moment of tipping over, which can be approximate to that at the moment of touchdown; $W_{H}$ refers to the increase of potential energy of the lander with the barycenter of the lander moving from the initial location at touchdown moment to the vertical plane containing centers of any two adjacent footpads; $W_{D}$ and $W_{H}$ can be calculated, respectively, by the following formulas:

(6) $$W_{D}=\frac{1}{2}m_{ld}\left(v_{v}^{2}+v_{h}^{2}\right)$$

(7) $$W_{H}=m_{ld}g^{\prime }∆h$$

where $m_{ld}$ is the mass of the main body, set as $1.372\times 10^{4}\;\mathrm{kg}$; $v_{v}$ and $v_{h}$ are the vertical velocity and horizontal velocity of the lander at the initial moment of touchdown respectively; $g^{\prime }$ is the gravitational acceleration on the lunar surface; and ∆h refers to the increase height of the barycenter of the lander corresponding to $W_{H}$.

Assuming that the main primary strut has no buffering stroke, ∆h can be estimated as follows:

(8) $$∆h=\sqrt{H_{0}^{2}+L_{v}^{2}}-H_{0}$$

According to Formulas (3)–(6), we can get:

(9) $$L_{v}\geq \frac{1}{2g^{\prime }}\sqrt{{\left(v_{v}^{2}+v_{h}^{2}\right)}^{2}+4g^{\prime }\left(v_{v}^{2}+v_{h}^{2}\right)H_{0}}$$

Actually, during the process of rolling over, a buffering stroke is produced to absorb partial impact energy, and thus the kinetic energy of the lander is reduced. Therefore, this estimate is conservative.

The impact energy needed to be absorbed can be decomposed into a vertical part $W_{v}$ and a horizontal part $W_{h}$. They can be obtained, respectively, by:

(10) $$W_{v}=\frac{1}{2}m_{ld}v_{v}^{2}+m_{ld}g^{\prime }∆H$$

(11) $$W_{h}=\frac{1}{2}m_{ld}v_{h}^{2}$$

Since extreme landing and landing surface conditions must be taken into consideration during the design of landing mechanisms, the buffer capacity of each primary strut can be assumed as follows:

(12) $$A_{p\_max}=a_{0}W_{v}$$

where $a_{0}$ is a constant considering landing uncertainties, and its value is generally less than 0.7. Meanwhile, it is assumed that each auxiliary strut is able to absorb impact energy in horizontal direction. Hence, the buffer capacity of each auxiliary strut can be obtained:

(13) $$B_{s\_max}=\frac{1}{2}m_{ld}v_{h}^{2}$$

Moreover, it is necessary to consider the limitation of the maximum response acceleration of the lander while landing. Thus, we have

(14) $$F\leq m_{ld}a_{max}$$

where $a_{max}$ is the maximum allowable response acceleration, and F is the buffering force.

According to Formulas (12) and (14), the minimum buffering stroke of the primary strut can be estimated:

(15) $$S_{p\_min}\approx A_{p\_max}/\left(F/n_{lg}\right)$$

where $n_{lg}$ is the number of landing mechanisms and obviously equals 4 here.

Above all, considering the size, strength and soft landing requirements synthetically, the materials and related parameters of landing mechanisms are as follows. All of the outer and inner cylinders of struts are made of aluminum alloy (7055) with elastic modulus of 70 GPa, poisson’s ratio of 0.3, density of $2850\mathrm{kg}/m^{3}$, and yield limit of 550 MPa. The geometric parameters are illustrated in Table 2.

### 3.4. Buffering Forces

The buffering force can be expressed as a function of relative stroke between the outer and inner cylinders. The buffering force of the primary strut is obtained by:

(16) $$F_{P,i}=\left\{\begin{array}{c} c_{v}\cdot F_{PH,i}\left(S_{P,i}\right)-f_{P,i},\;\;\;{\dot{S}}_{P,i}>0\;\mathrm{and}\;\left(S_{P,i}-S_{PR,i}\right)>0\\ \;\;\;\;\;\;\;\;\;\;\;\;\;\;\;\;-f_{P,i},\;\;\;\;\;\;\;\;\;\;\;\;\;\;\;{\dot{S}}_{P,i}\leq 0\;\mathrm{and}\;\left(S_{P,i}-S_{PR,i}\right)\leq 0 \end{array}\;\;\left(i=1,2,3,4\right)\;\right.$$

The buffering force of the auxiliary strut is obtained by:

(17) $$F_{Sj,i}=\left\{\begin{array}{c} c_{v}\cdot F_{SCj,i}\left(S_{Sj,i}\right)-f_{Sj,i},\;\;\;{\dot{S}}_{Sj,i}>0\;\mathrm{and}\;\left(S_{Sj,i}-S_{SCRj,i}\right)>0\\ c_{v}\cdot F_{STj,i}\left(S_{Sj,i}\right)-f_{Sj,i},\;\;\;{\dot{S}}_{Sj,i}<0\;\mathrm{and}\;\left(S_{STRj,i}-S_{Sj,i}\right)>0\\ \;\;\;-f_{Sj,i},\;\;\;\;\;\;\;\;\;\;\;\;\;\;\;\;\;\;\;\;\mathrm{others}\;\;\;\;\;\;\; \end{array}\;\left(j=1,2\right)\;\right.$$

where i denotes the number of landing legs, values 1 and 2 of j, respectively, represent the left auxiliary strut and right auxiliary struts from the outside view, $F_{P,i}$ and $F_{Sj,i}$ are the buffering forces of the main and auxiliary struts, respectively, and $c_{v}$ is the dynamic load coefficient which takes into account the effect of impact velocity. As is known, the initial impact velocity has little effect on the buckypaper’s buffering force as a function of stroke, and thus $c_{v}$ can be taken as 1. $S_{P,i}$ and $S_{Sj,i}$ represent the buffering stroke of the primary and auxiliary struts, respectively. ${\dot{S}}_{P,i}$ and ${\dot{S}}_{Sj,i}$ are the buffering velocity of the primary and auxiliary struts respectively, and they are defined to be positive for compression and negative for tension. $S_{PR,i}$, $S_{SCRj,i}$ and $S_{STRj,i}$ are permanent deformation of the buffer component within the primary strut, the compression buffer component within the auxiliary strut, and the tension buffer component within the auxiliary strut, respectively. $F_{PH,i}\left(S_{P,i}\right)$ is the compression force as a function of compression displacement of the buffer component within the primary strut. $F_{SCj,i}\left(S_{Sj,i}\right)$ and $F_{STj,i}\left(S_{Sj,i}\right)$ are the forces as a function of compression displacement of the buffer component for compression and tension buffer, respectively. $f_{P,i}$ and $f_{Sj,i}$ are the sliding friction between the inner and outer cylinders of the primary and auxiliary struts, respectively.

**Figure 6:**
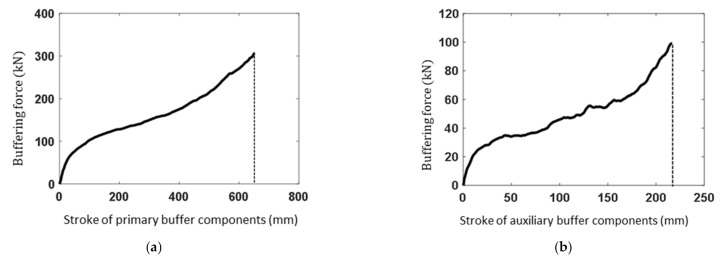
Buffering characteristics of buckypaper components within (**a**) primary and (**b**) auxiliary struts.

Since the compression strain rate has no obvious effect on the stress-strain relationship within the range of landing velocity (0–4 m/s), the buffering force as a function of the buffering stroke for a buckypaper-buffer component can be easily obtained based on the compression stress-strain relationship and the physical dimension. The area surrounded by the buffering force-stroke curve is just the energy absorption by the buffer component. Buffer components should meet the limitation of the physical dimensions within the struts and the energy absorption requirements. Meanwhile, buffering force is expected to be as small as possible. By comparing the buckypapers with five densities, the densities *ρ_bpp_* = 52.81 kg/m^3^ and *ρ_bpf_* = 91.01 kg/m^3^ have been selected for buffering materials of the primary struts and auxiliary struts, respectively. The physical dimensions of the buffer components are described in Table 3. Neglecting the rebound of the buckypaper, the buffering force as a function of the buffering stroke can be obtained based on the compression stress-strain relationship, shown in Figure 6. The mass of buckypaper components within one single landing mechanism can be acquired by:

(18) $$m_{bp}=\rho_{bpp}L_{bpp}\pi D_{bpp}^{2}/4+\rho_{bpf}L_{bpf}\pi \left(D_{bpf1}^{2}-D_{bpf2}^{2}\right)/4$$

where $D_{bpp}$ and $L_{bpp}$ are the initial diameter and length of the buckypaper component within the primary strut, respectively; and $D_{bpf1}$, $D_{bpf2}$, and $L_{bpf}$ are the initial outer diameter, inner diameter, and length of the buckypaper components for both tension and compression buffer. Finally, $m_{bp}$ is equal to 1.76 kg.

**Figure 7:**
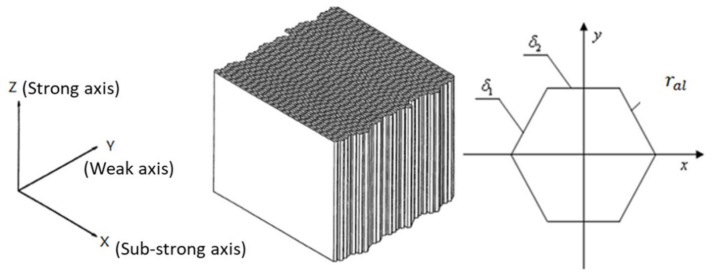
Diagram of aluminum honeycomb and the coordinate system.

**Figure 8:**
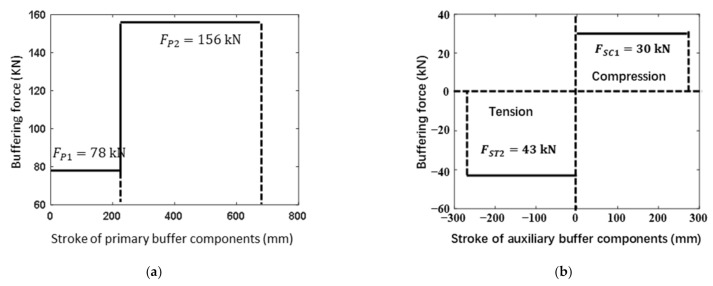
Buffering characteristics of aluminum honeycomb components within (**a**) primary and (**b**) auxiliary struts.

As is well known, aluminum honeycomb is the most commonly used energy absorption materials for landing mechanisms of a lunar lander. For the purpose of better understanding the buffering properties of buckypaper, aluminum honeycomb is also selected as an alternative buffering material for the lunar lander. The diagram of aluminum honeycomb and the definition of coordinate system are described in Figure 7. Aluminum honeycomb (abbreviated AH in Table 4) is an anisotropic material with parameters illustrated in Table 4, where $E_{xx}$, $E_{yy}$ and $E_{zz}$ are elasticity modulus in the corresponding directions, $G_{xy}$, $G_{zx}$, and $G_{yz}$ are shear modulus in the corresponding directions, and $\rho_{al}$ is the density. According to the mechanical properties of aluminum honeycomb material, the energy absorption process can be divided into three stages: elastic stage, plastic stage, and elastic stage of matrix material. The elastic stage of aluminum honeycomb material corresponds to the initial compression progress. Upon the compression load, elastic buckling occurs. Before the compression force reaches the peak, both the honeycomb and its matrix material have no plastic deformation. In the plastic stage, the aluminum honeycomb collapses under pressure and the plastic buckling deformation appears, resulting in a long platform of load. When the aluminum honeycomb is completely collapsed and compacted, it enters the elastic stage of the matrix material, causing the corresponding load rising sharply. Energy dissipation mainly depends on the plastic stage and can be obtained by the integral of compression load against compression stroke. The peak load during the elastic phase can be eliminated by applying a preload to the aluminum honeycomb. As is shown in Table 3, the aluminum honeycomb components have the same physical dimensions with the buckypaper ones, yet different available strokes. It is noted that the primary strut has two aluminum honeycomb components with different strengths. The relationship between the buffering force and the buffering stroke of the aluminum honeycomb components can be approximated as depicted in Figure 8. 

### 3.5. Forces upon the Main Body

According to Newton’s second law, the translational equation of the main body of the lander is:

(19) $$m_{ld}\left[\begin{array}{c} \ddot{X}\\ \ddot{Y}\\ \ddot{Z} \end{array}\right]=\left[\begin{array}{c} F_{X}-m_{ld}g^{\prime }\\ F_{Y}\\ F_{Z} \end{array}\right]$$

where X, Y, and Z are, respectively, the components of the displacement of the barycenter of the body in the inertial coordinate system, and $F_{X}$, $F_{Y}$, and $F_{Z}$ are, respectively, the components of the resultant force acting on the body induced by the landing mechanisms in the inertial coordinate system.

In the body coordinate system, the rotational Euler equation of the lander is:

(20) $$I_{X^{\prime }}{\dot{\omega }}_{X^{\prime }}-\omega_{Y^{\prime }}\omega_{z^{\prime }}\left(I_{Y^{\prime }}-I_{Z^{\prime }}\right)=N_{X^{\prime }}\;I_{Y^{\prime }}{\dot{\omega }}_{Y^{\prime }}-\omega_{Z^{\prime }}\omega_{X^{\prime }}\left(I_{Z^{\prime }}-I_{X^{\prime }}\right)=N_{Y^{\prime }}\;I_{Z^{\prime }}{\dot{\omega }}_{Z^{\prime }}-\omega_{X^{\prime }}\omega_{Y^{\prime }}\left(I_{X^{\prime }}-I_{Y^{\prime }}\right)=N_{Z^{\prime }}$$

where $I_{X^{\prime }}$, $I_{Y^{\prime }}$ and $I_{Z^{\prime }}$ are, respectively, the rotational inertias of the body about the $X^{\prime }$ axis, $Y^{\prime }$ axis, and $Z^{\prime }$ axes; $\omega_{X^{\prime }}$, $\omega_{Y^{\prime }}$ and $\omega_{Z^{\prime }}$ are, respectively, the corresponding components of angular velocity vector about each axis; and $N_{X^{\prime }}$, $N_{Y^{\prime }}$ and $N_{Z^{\prime }}$ are, respectively, the corresponding components of the moment applied by the landing mechanisms to the body.

The force applied on the body by the landing mechanisms can be expressed in the inertial coordinate system by:

(21) $$F_{X}=\sum_{i}\left(F_{PX,i}+F_{S1X,i}+F_{S2X,i}\right)\;F_{Y}=\sum_{i}\left(F_{PY,i}+F_{S1Y,i}+F_{S2Y,i}\right)\;F_{Z}=\sum_{i}\left(F_{PZ,i}+F_{S1Z,i}+F_{S2Z,i}\right)$$

where i=1,2,3,4, denoting the sequence number of the landing mechanisms; $F_{PX,i}$, $F_{PY,i}$ and $F_{PZ,i}$ are, respectively, the components of the force $F_{P,i}$ induced by the primary strut about each axis in the inertial coordinate system; similarly, $F_{S1X,i}$, $F_{S1Y,i}$ and $F_{S1Z,i}$ are respectively the components of the force $F_{S1,i}$ induced by the auxiliary strut with the number of i−1; $F_{S2X,i}$, $F_{S2Y,i}$ and $F_{S2Z,i}$ are, respectively, the components of the force $F_{S2,i}$ induced by the auxiliary strut with the number of i−2.

The corresponding momentum can be expressed in the body coordinate system by:

(22) $$N_{X^{\prime }}=\sum_{i}\left[\left(Y_{P,i}^{\prime }F_{PZ^{\prime },i}+Y_{S1,i}^{\prime }F_{S1Z^{\prime },i}+Y_{S2,i}^{\prime }F_{S2Z^{\prime },i}\right)-\left(Z_{P,i}^{\prime }F_{PY^{\prime },i}+Z_{S1,i}^{\prime }F_{S1Y^{\prime },i}+Z_{S2,i}^{\prime }F_{S2Y^{\prime },i}\right)\right]\;N_{Y^{\prime }}=\sum_{i}\left[\left(Z_{P,i}^{\prime }F_{PX^{\prime },i}+Z_{S1,i}^{\prime }F_{S1X^{\prime },i}+Z_{S2,i}^{\prime }F_{S2X^{\prime },i}\right)-\left(X_{P,i}^{\prime }F_{PZ^{\prime },i}+X_{S1,i}^{\prime }F_{S1Z^{\prime },i}+X_{S2,i}^{\prime }F_{S2Z^{\prime },i}\right)\right]\;N_{Z^{\prime }}=\sum_{i}\left[\left(X_{P,i}^{\prime }F_{PY^{\prime },i}+X_{S1,i}^{\prime }F_{S1Y^{\prime },i}+X_{S2,i}^{\prime }F_{S2Y^{\prime },i}\right)-\left(Y_{P,i}^{\prime }F_{PX^{\prime },i}+Y_{S1,i}^{\prime }F_{S1X^{\prime },i}+Y_{S2,i}^{\prime }F_{S2X^{\prime },i}\right)\right]$$

where i still denotes the sequence number of the landing mechanisms; $F_{PX^{\prime },i}$, $F_{PY^{\prime },i}$, and $F_{PZ^{\prime },i}$ are, respectively, the components of $F_{P,i}$ about each axis of the body coordinate system; $F_{S1X^{\prime },i}$, $F_{S1Y^{\prime },i}$, $F_{S1Z^{\prime },i}$, $F_{S2X^{\prime },i}$, $F_{S2Y^{\prime },i}$ and $F_{S2Z^{\prime },i}$ are, respectively, the components of $F_{S1,i}$ and $F_{S2,i}$ about each axis of the body coordinate system. $X_{P,i}^{\prime }$, $Y_{P,i}^{\prime }$ and $Z_{P,i}^{\prime }$ are the coordinates of the connection point between the primary strut and the body of the lander in the body coordinate system; $X_{S1,i}^{\prime }$, $Y_{S1,i}^{\prime }$ and $Z_{S1,i}^{\prime }$ are the coordinates of the connection point between the auxiliary strut with the number of i−1 and the main body in the body coordinate system; and $X_{S2,i}^{\prime }$, $Y_{S2,i}^{\prime }$ and $Z_{S2,i}^{\prime }$ are the coordinates of the connection point between the auxiliary strut with the number of i−2 and the main body in the body coordinate system.

### 3.6. Contact Force between Footpads and the Lunar Surface

The real interaction between the footpads and the lunar surface is rather complicated. In multi-body dynamics, it can be simplified to be a normal force and a tangential force. For the normal force, a nonlinear spring damping model is used to simulate the impact of the footpads on the lunar surface. The tangential force includes the following two parts. One is the frictional force caused by the slipping between the footpads and the lunar surface. And the other is the bulldozing force applied on the side of the footpads by the sinking of the footpads. This frictional force is modeled by coulomb friction and the bulldozing force is a function depending on the depth and velocity of the sinking of the footpads. Hence, the normal force and the tangential force can be calculated by:

(23) $$F_{N}=K_{N}\delta_{L}^{e_{N}}+C_{N}{\dot{\delta }}_{L}$$

(24) $$F_{T}=\mu_{T}F_{N}+K_{T}\delta_{L}^{e_{T}}{\dot{\delta }}_{L}$$

where $K_{N}$, $e_{N}$ and $C_{N}$ are respectively the stiffness, nonlinear exponent and damping coefficient, all related about the sinking depth $\delta_{L}$; $K_{T}$ and $e_{T}$ are bulldozing coefficients related to $\delta_{L}$; $\mu_{T}$ is the friction coefficient between the footpads and the lunar surface, which is set as 0.4.

### 3.7. Initial Landing Conditions and Landing Cases

**Figure 9:**
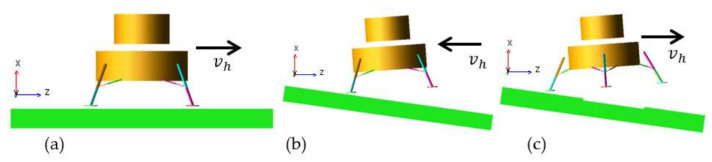
Diagrams of three severe landing case. (**a**) High-overloading case; (**b**) Easily overturning case; (**c**) Long-stroke case.

All the components of the lander except the energy absorption components are approximately regarded to be rigid. The soft landing dynamics simulations of the lander are implemented based on the popular commercial software of MSC. Adams. According to current references [3,4], the initial landing conditions of a lunar lander is summarized as Table 5. To analyze the soft-landing dynamic properties and validate the design reasonability, three severe landing cases are selected based on amounts of simulations, including the high-overloading case (Case 1), the easily overturning case (Case 2) and the long-stroke case (Case 3). According to the reference [4], they are depicted in Table 6 in detail and exhibited in Figure 9.

## 4. Simulation Results and Discussions

### 4.1. Overload Response of the Mass Center

**Figure 10:**
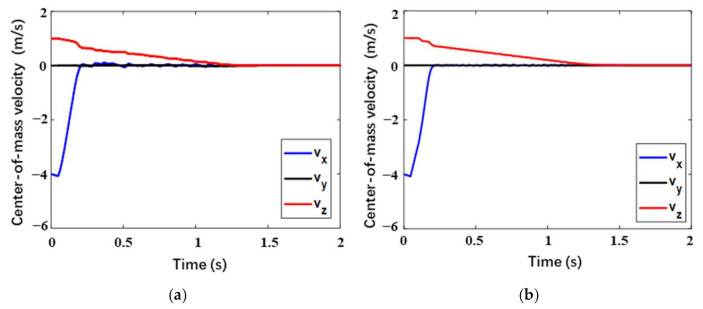
Center-of-mass velocity of lunar lander in Case 1 within (**a**) buckypaper and (**b**) aluminum honeycomb.

In this section, the high-overloading case (Case 1) corresponding to the limitation of the center-of-mass overload is mainly focused on. For better analyzing the overload response, the center-of-mass velocity response is firstly observed. Considering the overload bearing capacity of the astronauts, the center-of-mass acceleration a of the manned lunar lander is generally required to be less than 5 g, that is, $a\leq a_{max}=5\;g$. As is represented in Figure 10, for the two landers with different buffering materials, both the velocities in the X-axis direction (vertical velocity) increase slightly during the short free-fall stage of the initial 0.05 s. After the footpads touching the lunar surface, it decreases to almost zero within 0.2 s. The velocities in Z-axis direction for the two landers are not quite the same. The difference is that the buckypaper-buffering lander needs 1.24 s to mitigate this horizontal velocity completely while the aluminum honeycomb-buffering lander needs 1.3 s. Additionally, the velocity in Y-axis direction maintains zero during the whole landing progress. Above all, the buckypaper-buffering lander reaches the static state earlier than the aluminum-buffering one by 0.06 s.

**Figure 11:**
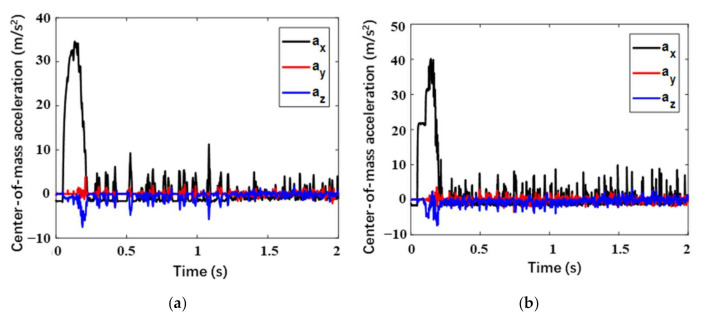
Center-of-mass acceleration of lunar lander in Case 1 within (**a**) buckypaper and (**b**) aluminum honeycomb.

In this load case, corresponding to the two buffering materials, the center-of-mass accelerations of the two landers as a function of time are described in Figure 11. It is necessary to declare that all the acceleration responses adopt low passing filter of 80 MHz. It can be seen that the two acceleration responses are analogous. During the initial 0.05 s, the lander is in free fall, therefore, the center-of-mass acceleration is just the local gravitational acceleration of the lunar surface. When the footpads touch the lunar surface, the acceleration in the X-axis direction will rapidly decrease to zero and then points to the opposite direction, followed by a quick increase and a subsequent decrease in the module. Due to the influence of the horizontal velocity, a low acceleration in the Z-axis also appears. In the Y-axis direction, the acceleration is almost zero. It can be seen form Figure 10 that the high overload mainly occurs on the X-axis. The maximum overload of the buckypaper-buffering lander is 3.46 g while that of the aluminum honeycomb-buffering lander is 4.00 g. During the buffering progress, a stepped leapfrog phenomenon appears in the overload response of the aluminum honeycomb-buffering lander. It is due to the fact that there are two stages of aluminum honeycomb components with different strengths in the primary strut, and the strong one begins to work only when the weak one is compacted. However, for the buckypaper-buffering lander, the overload response is relatively smooth. Moreover, the high overload the lander suffers mainly occurs within the initial 0.2 s. Over the period of 0.2 s to 1.3 s, the rest slight accelerations in both X and Y directions will decrease to zero gradually.

In addition, the important results about the center-of-mass velocity and overload responses for the three cases are demonstrated in Table 7. For both the two landers, the maximum center-of-mass accelerationss are lower than the maximum allowable value of 5 g. Both in Case 1 and Case 2, it spends shorter time finishing the landing process for the buckypaper-buffering lander than the aluminum honeycomb-buffering one, and the overload of the former is lower than that of the latter. However, the conclusions are contrary in Case 3.

### 4.2. Overturning Resistance Capability

During the whole landing process, in order to prevent the lander from overturning, the center of mass of the lander should not exceed the “stability walls” which are formed by the vertical planes extended from the “stability polygon”. This is to say that the center of mass of the lander should always lies within the space enclosed by the “stability walls”. Therefore, it is necessary to monitor the distances between the center of mass of the lander and the four stability walls, respectively. Suppose the centers of all the footpads are $B_{i}$ (i=1, 2, 3, 4, corresponding to the sequence number of the landing mechanism), the center of mass of the lander is $B_{0}$, the projection of the center of mass on the “stability wall ij” which involves centers of two adjacent footpads $B_{i}$ and $B_{j}$ is $B_{0}^{ij}$, the distance vector from $B_{0}$ to $B_{j}$ is $B_{0j}$, the distance vector from $B_{i}$ to $B_{j}$ is $B_{ij}$, and the gravity vector of the lunar is $g^{\prime }$, the distance $B_{0\_ij}$ from the center of mass to the stability wall involving $B_{i}$ and $B_{j}$, also called “stability distance ij”, can be calculated by:

(25) $$B_{0\_ij}=\frac{B_{0j}\cdot \left(g^{\prime }\times B_{ij}\right)}{\left|g^{\prime }\times B_{ij}\right|}ij=12,\;23,\;34,\;41$$

If $B_{0\_ij}>0$, the center of mass is located within the space enclosed by the stability walls. If $B_{0\_ij}<0$, the center of mass is located outside the stability walls. If $B_{0\_ij}=0$, the center of mass is just located on the stability walls.

**Figure 12:**
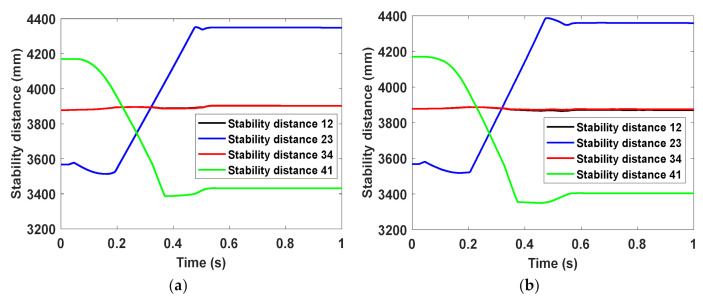
Distances from the center of mass to the stability walls in Case 2 within (**a**) buckypaper and (**b**) aluminum honeycomb.

In this section, the easily overturning case (Case 2) corresponding to the limitation of the stability distance is mainly focused on. The four stability distances are, respectively, recorded in Figure 12. It can be seen that the simulation results about the stability distances are semblable for the two landers and the landing process has a great influence on stability distance 23 and stability distance 41. During the initial landing stage, only the upslope Leg 2 and Leg 3 touch the lunar soil, and thus Leg 2 and Leg 3 begin to buffer. Due to the influence of horizontal velocity, the stability distance 23 decreases first and then increases until the whole landing is finished. Yet, the stability distance 41 keeps decreasing until Leg 1 and Leg 4 touch the lunar surface, followed by a slight increase as a result of the lateral slipping of the footpads of Leg 1 and Leg 4. Besides, the stability distance 12 and stability distance 34 are nearly identical all the time due to the symmetry of the lander about the xz plane and change slightly over time during the landing process. It also can be seen from the figure that the minimum stability distance is stability distance 41. The corresponding value is 3386 mm and 3349 mm for the buckypaper-buffering lander and aluminum honeycomb-buffering, respectively, indicating a better stability for the former to some extent.

In addition, the minimum stability distances of the two landers in the three severe cases are listed in Table 8. It can be found that the minimum stability distance of the buckypaper-buffering lander is larger than that of the aluminum honeycomb-buffering lander in both Case 2 and Case 3, while the former is slightly smaller than the latter in Case 1. In addition, considering that if the distance between the bottom of the lander and the lunar surface (also called “bottom-lunar distance” for short) is too small, the bottom structure of the lander is likely to collide with rocks on the lunar surface while landing, probably causing turnover of the lander. Hence, bottom-lunar distance is required to be not less than a certain value $D_{cr}$, and herein $D_{cr}=300\;\mathrm{mm}$. The bottom-lunar distance versus time during the whole landing process can be recorded, and then the minimum values for the two landers in three cases are also summarized in Table 8. It can be found that for the two landers the bottom-lunar distances are adequate in all the cases. In both Case 1 and Case 3, the minimum values for the two landers are quite close, and in Case 2 that of the buckypaper-buffering lander is slightly smaller than the that of the other lander.

### 4.3. Buffering Stroke

While landing, the landing mechanisms absorb the impact energy by compressing the buffer components within them, resulting in buffering strokes of the primary struts and the auxiliary struts. The buffering strokes of the primary strut and auxiliary strut, denoted by $S_{P,i}$ and $S_{Sj,i}$ respectively, should not be larger than the designed maximum strokes, denoted $S_{P,max}$ and $S_{S,max}$, respectively. Herein, for aluminum honeycomb buffers, $S_{P,i}\leq S_{P,max}=675\;\mathrm{mm}$, and $S_{Sj,i}\leq S_{S,max}=270\;\mathrm{mm}$. For buckypaper buffers, $S_{P,i}\leq S_{P,max}=696\;\mathrm{mm}$, and $S_{Sj,i}\leq S_{S,max}=231\;\mathrm{mm}.$ In this section, the long-stroke case (Case 3) is mainly focused on. 

**Figure 13:**
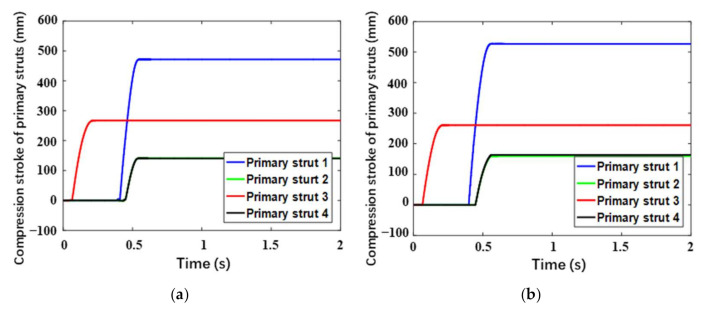
Compression stroke of primary struts versus time for (**a**) buckypaper-buffering lander and (**b**) aluminum honeycomb-buffering lander.

**Figure 14:**
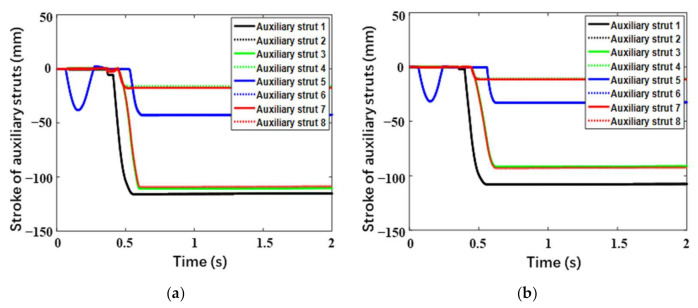
Stroke of auxiliary struts versus time for (**a**) buckypaper-buffering lander and (**b**) aluminum honeycomb-buffering lander.

In this section, the long stroke case (Case 3) corresponding to the limitation of the buffering stroke is mainly focused on. Figure 13 and Figure 14, respectively, describe the buffering strokes versus time for the primary struts and auxiliary struts. Generally, the change tendencies and laws are approximate for the two landers. Primary Strut 2 and Primary Strut 4 always have the same buffering strokes due to their symmetry. At the initial stage, compression buffering firstly occurs in Primary Strut 3 of upslope Leg 3, and simultaneously the corresponding tension buffering appears in Auxiliary Strut 5 and Auxiliary Strut 6. Due to the concaves below Leg 2 and Leg 3, Leg 1, located downhill, becomes the second landing leg to touch the lunar surface and buffers, quickly followed by Legs 2 and Leg 4. Thereinto, Primary Strut 1 has the largest compression stroke, followed by Primary Sturt 3, and Primary Strut 2 and Primary Strut 4 have the minimum compression strokes with the same value. Besides, Auxiliary Strut 1 and Auxiliary Strut 2 have the largest tension strokes with the same value, followed by Auxiliary Strut 3 and Auxiliary Strut 8 with the second-largest ones, and Auxiliary Strut 1 and Auxiliary Strut 2 with the minimum ones. Especially, a second tension buffering happens on Auxiliary Strut 5 and Auxiliary Strut 6 in Leg 3, during which the tension length is only 1–2 mm owing to the plastic deformation of buffering components. Above all, the maximum compression strokes of the primary struts for the buckypaper-buffering lander and the aluminum honeycomb-buffering lander are, respectively, 471.3 mm and 526.9 mm, the former smaller than the latter by 55.6 mm. The largest tension strokes of the auxiliary struts for the buckypaper-buffering lander and the aluminum honeycomb-buffering lander are, respectively, 115.7 mm and 108.0 mm, the latter slightly larger than the latter. Besides, it can be found that all the auxiliary struts hardly have any compression stroke.

In addition, Table 9 concludes the maximum buffering stoke of the primary struts and auxiliary struts, respectively, for the two landers in the three cases. It can be observed that all the maximum practical buffering strokes can meet the design requirements. What’s more, for each case, the maximum buffering stroke of the primary struts for the buckypaper-buffering lander is always shorter than that of the aluminum honeycomb-buffering lander. Yet, for the maximum buffering strokes of the auxiliary struts, there is little difference between the two landers, only 7.7 mm for the maximum difference.

## 5. Conclusions

In this work, a coarse-grained molecular dynamics method is used to implement a series of simulations about uniaxial compression of buckypapers with different densities. The results indicate that Young’s modulus of a buckypaper increases with its density, accompanied with the improvement of its energy absorption capacity. Besides, the compression rate within the range of the landing velocity has little influence on the stress-strain relationship, and thus the influence of the landing velocity on the relationship between the buffering force and buffering stroke can be ignored. That is, the buffering force as a function of the buffering stroke can be substituted by the quasi-static compression stress as a function of compression strain. Two energy absorption materials, buckypaper and aluminum honeycomb, were, respectively, selected for the parametric design of two landing mechanisms, thus, respectively, establishing two soft-landing multibody dynamics models of two landers with a difference only in their energy absorption materials. Then a series of simulations are implemented to explore the landing properties of the two landers under three severe cases. It is found that both of the two landers are able to land on the lunar surface smoothly and safely under all the three cases. In the high-overloading case, during the whole landing process, the highest center-of-mass overloads of the buckypaper-buffering lander and the aluminum honeycomb-buffering lander are, respectively, 3.46 g and 4.00 g, both of which are lower than the allowable value 5 g. What is important is that, compared with the aluminum honeycomb components, the buckypaper components cutdown the highest overload by 0.54 g, which can improve the comfort of astronauts and reduce the loads induced on related structure and devices. In the easily overturning case, the barycenters of both the two landers are always located within the range of the stability walls and keep far away from the walls. The minimum stability distances are 3386 mm and 3349 mm, respectively, for the buckypaper-buffering lander and aluminum honeycomb-buffering lander. Meanwhile the bottoms of the two landers always remain adequate distance from the lunar surface, 471.3 mm, and 526.9 mm, respectively, for the buckypaper-buffering lander and aluminum honeycomb-buffering lander. In the long-stroke case, the maximum compression strokes of the primary struts for both the two landers are within their required stroke ranges, 471.3 mm and 526.9 mm, respectively, for the buckypaper-buffering lander and aluminum honeycomb-buffering lander. The above differences of the results between the two landers are probably due to the difference between the curves of buffering force versus buffering stroke. Remarkably, compared with the aluminum honeycomb components, the buckypaper can reduce the total mass of the buffering components by 52.71%. In other words, the mass of the lander can be decreased by 8.14 kg, indicating less fuel and less cost to a great extent. This is owing to the buckypaper’s much lower density than the aluminum honeycomb. In summary, compared to the aluminum honeycomb-buffering lander, the buckypaper-buffering lander has not only better landing performance, but also much lighter mass. In spite of the fact that all of these findings are obtained in silico, this study can shed lights on the superiority of buckypapers in the aspect of crashworthiness while applied in a soft-landing mechanism of a manned lunar lander and is believe that our results will stimulate more relevant practical work.

## Figures and Tables

**Table 1:** Potential parameters for the coarse-grained model of (5,5) SWCNT.

Parameters	(5,5) SWCNT
Equilibrium bead distance, $r_{b0}$ (Å)	10
Stretching constant, $k_{b}$ ($\mathrm{kcal}/(\mathrm{mol}\cdot {Å}^{2})$)	1000
Equilibrium angle, $\theta_{0}$ (°)	180
Bending constant, $k_{a}$ ($\mathrm{kcal}/(\mathrm{mol}\cdot {\mathrm{rad}}^{2})$)	14,300
vdW distance, σ (Å)	9.35
vdW energy, ε (kcal/mol)	15.10

**Table 2:** Parameters of landing mechanisms.

Parameters of Landing Mechanisms		Value (mm)
$H_{0}$		3318
$L_{h}$		1443
$L_{v}$		5484
Primary struts	Initial length	3506
	Outer diameter of outer cylinders	216
	Outer diameter of inner cylinders	180
	Wall thickness of outer/inner cylinders	3
	Overlap length of outer-inner cylinders	833
Auxiliary strut	Initial length	1774
	Outer diameter of outer cylinders	98
	Outer diameter of inner cylinders	50
	Wall thickness of outer/inner cylinders	3
	Overlap length of outer-inner cylinders	512

**Table 3:** Parameters of buffer components.

Parameters of Buffer Components (mm)		Buckypaper Components	Aluminum Honeycomb Components			
Buffer components of primary struts	Diameter,$\;D_{bpp}$	204		204		204
	Length, $L_{bpp}$	870	Strong:	580	Weak:	290
	Maximum stroke, $S_{PR,max}$	696		450		225
Tension/compression components of auxiliary struts	Outer diameter, $D_{bpf1}$	94	94			
	Inner diameter, $D_{bpf2}$	50	50			
	Length, $L_{bpf}$	289	289			
	Maximum stroke, $S_{SR,max}$	231	250			

**Table 4:** Parameters of aluminum honeycomb materials.

Aluminum Honeycomb Components	$E_{xx}\;(\mathrm{MPa})$	$E_{yy}\;\left(MPa\right)$	$E_{zz}\;\left(MPa\right)$	$G_{xy}\;\left(MPa\right)$	$G_{zx}\;\left(MPa\right)$	$G_{yz}\;\left(MPa\right)$	$\rho \;\left(g/cm^{3}\right)$
Primary strut-Weak AH	1.01	1.02	2.29	0.59	286	573	0.0912
Primary strut-Strong AH	3.42	3.47	4.59	2.03	429	860	0.1296
Auxiliary strut-Compression AH	3.42	3.47	6.19	2.03	0.75	860	0.1344
Auxiliary strut-Tension AH	11.54	11.70	8.88	6.85	645	1291	0.1984

**Table 5:** Initial landing conditions.

Requirements	Initial Value
Vertical touchdown velocity(m/s)	−4~0
Horizontal touchdown velicty(m/s)	−1~1
Landing surface slope(deg)	−8~8
Yaw angle(deg)	−45~45
Pitch angle(deg)	−4~4
Depth of concave(mm)	0~200

**Table 6:** Landing cases.

Requirements	Case 1	Case 2	Case 3
Vertical touchdown velocity $V_{x}$ (m/s)	−4	−4	−4
Horizontal touchdown velicty $V_{y}$ (m/s)	0	0	0
Horizontal touchdown velicty $V_{z}$ (m/s)	1	−1	1
Landing surface slope α (deg)	0	−8	−8
Yaw angle φ (deg)	45	45	0
Pitch angle ∅ (deg)	0	4	4
Depth of concave $d_{c}$ (mm)	0	0	200 (Footpads 2, 4)

**Table 7:** Important data about the center-of-mass overload responses.

Cases	Landers	Time for the Whole Landing (s)	Maximum Overload (g)
Case 1	Buckypaper	1.24	3.46
	Aluminum honeycomb	1.30	4.00
Case 2	Buckypaper	0.68	2.66
	Aluminum honeycomb	0.92	2.76
Case 3	Buckypaper	3.60	2.71
	Aluminum honeycomb	3.21	2.62

**Table 8:** Important data about key distances related to overturning resistance capability.

Cases	Landers	Minimum Stability Distance (mm)	Minimum Bottom-Lunar Distance (mm)
Case 1	Buckypaper	3789	692
	Aluminum honeycomb	3798	695
Case 2	Buckypaper	3386	694.8
	Aluminum honeycomb	3349	721.5
Case 3	Buckypaper	3384	794.6
	Aluminum honeycomb	3336	794.1

**Table 9:** Maximum buffering stroke of the primary struts and auxiliary struts in three cases.

Cases	Landers	Maximum Stoke of Primary Struts (mm)	Maximum Stoke of Auxiliary Struts (mm)
Case 1	Buckypaper	280.5	112.9
	Aluminum honeycomb	289.1	118.6
Case 2	Buckypaper	314.6	110.5
	Aluminum honeycomb	332.6	109.4
Case 3	Buckypaper	471.3	115.7
	Aluminum honeycomb	526.9	108.0

## Data Availability

The data used to support the findings of this study are included within the article.
